# Short-term effects of an SGLT2 inhibitor on divalent ions in autosomal dominant polycystic kidney disease: a randomized crossover trial

**DOI:** 10.1093/ckj/sfag349

**Published:** 2026-10-02

**Authors:** Lorena Roth, Dusan Harmacek, Thomas Schachtner, Britta George, Cornelia Hofmann, Argyrios D Georgalis, Reto M Venzin, Patrick Hofmann, Thomas H Fehr

**Affiliations:** Department of Internal Medicine, Cantonal Hospital Graubuenden, Chur, Switzerland; Department of Nephrology, University Hospital Zurich, Zurich, Switzerland; Department of Nephrology, University Hospital Zurich, Zurich, Switzerland; University of Zurich, Zurich, Switzerland; Department of Nephrology, University Hospital Zurich, Zurich, Switzerland; University of Zurich, Zurich, Switzerland; Institute of Computational Life Sciences, Zurich University of Applied Sciences, Waedenswil, Switzerland; Department of Internal Medicine, Cantonal Hospital Graubuenden, Chur, Switzerland; Division of Nephrology, Cantonal Hospital Graubuenden, Chur, Switzerland; Department of Internal Medicine, Cantonal Hospital Graubuenden, Chur, Switzerland; Division of Nephrology, Cantonal Hospital Graubuenden, Chur, Switzerland; Department of Internal Medicine, Cantonal Hospital Graubuenden, Chur, Switzerland; Renal Division, Department of Medicine, Mass General Brigham, and Harvard Medical School, Boston, USA; Harvard T.H. Chan School of Public Health, Boston, USA; Department of Internal Medicine, Cantonal Hospital Graubuenden, Chur, Switzerland; University of Zurich, Zurich, Switzerland

**Keywords:** autosomal dominant polycystic kidney disease, calcium, magnesium, phosphate, SGLT2 inhibitor

## Abstract

**Background:**

Sodium–glucose cotransporter 2 (SGLT2) inhibitors are a cornerstone of chronic kidney disease therapy, reducing disease progression, cardiovascular events, and mortality. Autosomal dominant polycystic kidney disease (ADPKD) patients were excluded from major trials, and data on electrolyte handling with SGLT2 inhibitors, in combination with tolvaptan, are lacking.

**Methods:**

SIDIA was a randomized, double-blind, placebo-controlled, two-period crossover trial in 15 adults with ADPKD (mean estimated glomerular filtration rate (eGFR) 68 ml/min/1.73 m²; median age 43 years). Participants received empagliflozin 10 mg or placebo for 14 days, separated by a 2-week washout. The primary endpoint was 24 h urinary and fractional excretion of phosphate, calcium, and magnesium. Exploratory secondary endpoints included markers of kidney stone propensity, inflammation, vitamin D metabolism, and tolerability. Treatment effects were estimated using generalized linear mixed models. Given the small sample size, analyses were exploratory and focused on effect estimation.

**Results:**

For the primary endpoint, neither 24 h urinary nor fractional excretion of phosphate, calcium, or magnesium differed between empagliflozin and placebo. Empagliflozin lowered serum uric acid (–26%), increased serum magnesium (+5.7%) and urinary citrate (+78.6%), and reduced blood pressure (–4/–7 mmHg). An expected decline in eGFR (–7.1%) was observed, with no major short-term safety signals observed. Copeptin levels increased with empagliflozin (+26%).

**Conclusions:**

In this exploratory mechanistic trial, no substantial short-term difference in urinary or fractional excretion of divalent ions were detected with empagliflozin. The observed changes in serum magnesium, urinary citrate, uric acid, and copeptin are hypothesis-generating and require confirmation in larger, longer-term studies.

## Graphical Abstract

**Graphical Abstract:**
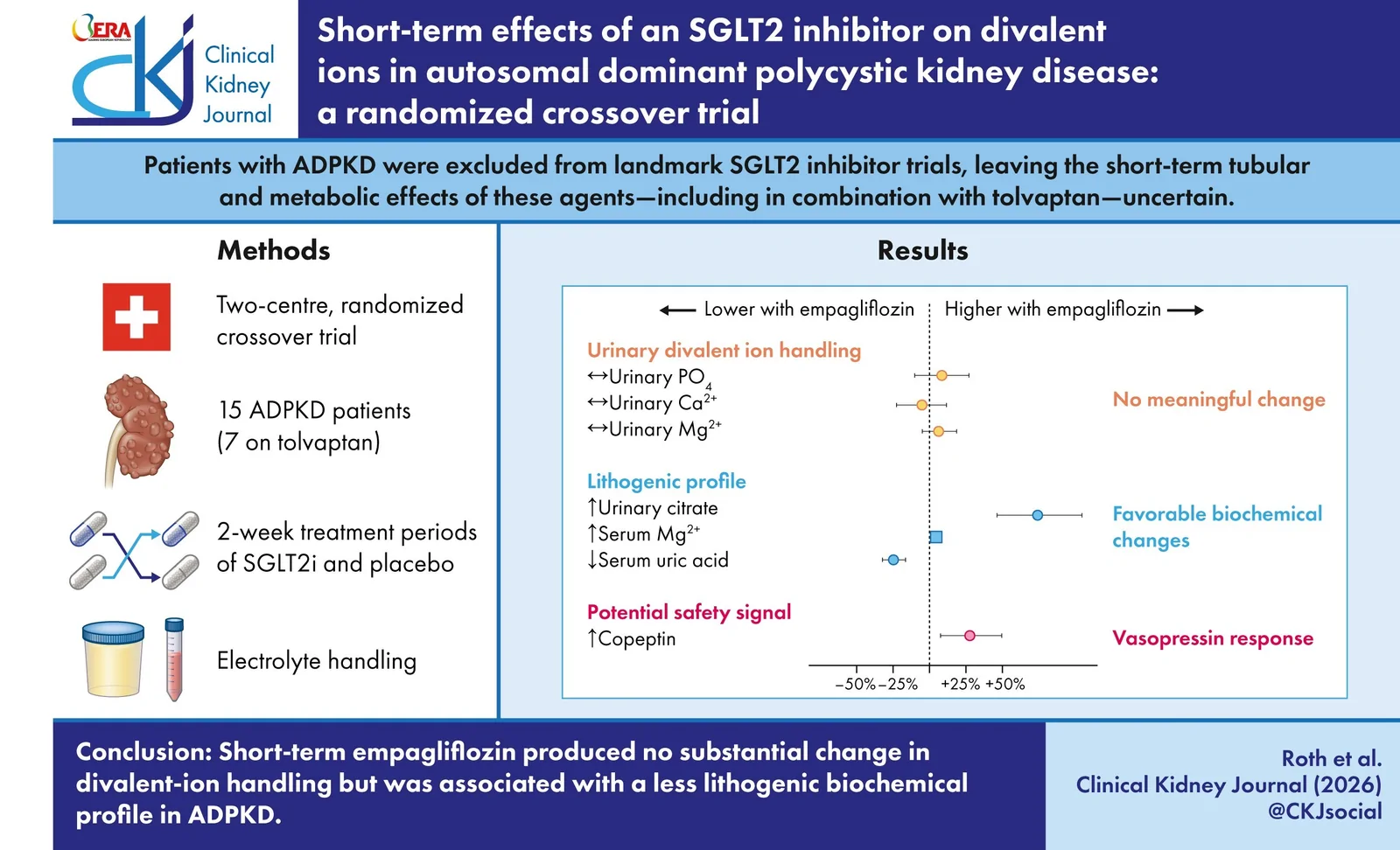

KEY LEARNING POINTS **What was known:**

- Patients with autosomal dominant polycystic kidney disease (ADPKD) were excluded from major sodium–glucose cotransporter 2 (SGLT2) inhibitor trials, leaving uncertainty regarding their metabolic and tubular effects in this population.
- ADPKD is associated with altered phosphate handling, elevated fibroblast growth factor 23 (FGF23) levels, and risk of nephrolithiasis.
- SGLT2 inhibitors increase serum magnesium and may reduce kidney stone risk in chronic kidney disease and diabetes.

**This study adds:**

- In this small crossover trial, no substantial short-term differences in urinary or fractional excretion of phosphate, calcium, or magnesium were detected with empagliflozin.
- Empagliflozin was associated with higher urinary citrate and serum magnesium, and lower serum uric acid, consistent with a less lithogenic profile.
- Copeptin increased with empagliflozin, identifying a potential vasopressin-related response requiring longer-term evaluation.

**Potential impact:**

- These hypothesis-generating findings provide preliminary mechanistic data and effect-size estimates to inform future trials.
- Larger, longer-term studies are needed to determine the clinical efficacy and safety of SGLT2 inhibition in ADPKD, including its effects on nephrolithiasis and disease progression.

## INTRODUCTION

Autosomal dominant polycystic kidney disease (ADPKD) is the most frequent hereditary kidney disease and a major cause of kidney failure worldwide, affecting up to 12 million people [1]. It is characterized by progressive cyst growth, kidney enlargement, and gradual loss of kidney function [2–4]. ADPKD is associated with hepatic cysts, vascular abnormalities, hypertension, nephrolithiasis, and cyst infections [3].

Current therapy for ADPKD is largely supportive, emphasizing optimal blood pressure control and cardiovascular risk reduction. The vasopressin V_2_-receptor antagonist tolvaptan is the only approved disease-modifying treatment that slows cyst growth and kidney function decline [5, 6]. However, its use is limited by hepatic toxicity, aquaretic side effects, and cost [7]. A recent randomized trial showed that high water intake alone did not slow kidney-volume growth, underscoring the need for additional or complementary therapeutic approaches [8, 9].

Sodium–glucose cotransporter 2 (SGLT2) inhibitors provide broad cardiorenal protection across diabetes, heart failure, and chronic kidney disease (CKD) of diverse etiologies. Their nephroprotective actions—mediated by tubuloglomerular feedback and metabolic re-programming—reduce intraglomerular pressure and oxidative stress [10, 11]. Yet, patients with ADPKD were excluded from pivotal trials such as DAPA-CKD and EMPA-KIDNEY, and evidence in this population remains scarce [12, 13].

In ADPKD, potential disease-specific benefits include attenuation of hyperfiltration and favorable modulation of metabolic pathways implicated in cystogenesis, whereas concerns include vasopressin activation, uncertain effects on cyst growth, and additive polyuria when combined with tolvaptan [14–17].

Beyond established nephroprotective and metabolic effects, SGLT2 inhibitors influence mineral metabolism. Early reports linked canagliflozin to a mild increase in serum phosphate and fibroblast growth factor 23 (FGF23), raising concerns about bone health; however, large cohort studies have not confirmed sustained effects or higher fracture risk [18–21]. In addition, SGLT2 inhibitors are consistently associated with higher serum magnesium concentrations and a lower risk of kidney stone formation, possibly reflecting favorable changes in tubular electrolyte handling and urinary citrate excretion [22, 23]. In ADPKD, phosphate metabolism is already altered by elevated FGF23 and renal phosphate wasting, yet how SGLT2 inhibition affects these abnormalities remains unknown [24]. Accordingly, phosphate, calcium, and magnesium were selected as primary endpoints to capture potential SGLT2 inhibitor-induced changes in proximal tubular transport and downstream mineral handling.

Despite growing interest in SGLT2 inhibition for ADPKD, its effects on electrolyte and mineral metabolism have not been studied. We therefore conducted a randomized, placebo-controlled, crossover trial (SIDIA) to evaluate the short-term impact of empagliflozin on the handling of divalent ions—phosphate, magnesium, and calcium—in patients with ADPKD, including those receiving concomitant tolvaptan.

## MATERIALS AND METHODS

### Study design and oversight

This was an investigator-initiated, randomized, double-blind, placebo-controlled, two-period crossover mechanistic trial conducted at the Cantonal Hospital Graubuenden (KSGR, Chur) and the University Hospital Zurich (USZ), Switzerland. The protocol was approved by the local ethics committee (BASEC-2024-00070), and all participants provided written informed consent before any study procedure. The trial was registered prospectively at the Swiss National Clinical Trials Portal (SNCTP000005836) and ClinicalTrials.gov (NCT06435858). Funding sources had no role in study design, data collection, analysis, or reporting.

The trial was conducted and reported in accordance with the CONSORT 2025 statement and for randomized crossover trials [25].

### Participants

Adults with a confirmed diagnosis of ADPKD and stable kidney function were eligible for enrolment; use of tolvaptan according to standard clinical care was permitted. Concomitant medication was required to remain stable throughout the study period (Table S1). Key exclusion criteria were pregnancy, diabetes mellitus type 1, uncontrolled hypertension, the use of thiazide diuretics, active infection, or any contraindication to SGLT2 inhibition. Participants were recruited at two sites, the KSGR and USZ, both in Switzerland. The complete list of inclusion and exclusion criteria is provided in *Supplementary Material.*

### Randomization, masking, and interventions

Participants were randomized in a 1:1 ratio to treatment sequence (empagliflozin -> placebo or placebo -> empagliflozin) using a web-based randomization tool. Empagliflozin 10 mg or matched placebo was administered once daily for 2 weeks in each period, separated by a 2-week washout.

The trial used a double-blind design. Participants and treating physicians were blinded to treatment allocation, whereas laboratory staff and the biostatistician remained unblinded; laboratory blinding was not feasible because glucosuria reliably identifies SGLT2 inhibition.

Due to a site-level dispensing irregularity, four participants received the same treatment in both periods (two empagliflozin–empagliflozin and two placebo–placebo). These participants were retained in the per-protocol analysis as prespecified. Comprehensive details on randomization procedures, adherence assessment, handling of protocol deviations, and analytical handling of the allocation irregularity are available in the *Supplementary Material.*

### Outcomes

The primary outcome was the effect of empagliflozin versus placebo on renal tubular handling of divalent ions—phosphate, magnesium, and calcium—assessed by 24-h urinary excretion and corresponding fractional excretion.

Secondary exploratory outcomes include tubular handling of other electrolytes (sodium, potassium, and chloride) and markers of mineral and bone metabolism (parathyroid hormone [PTH], FGF23, and 25-hydroxy-vitamin D), additional urinary parameters (volume, pH, oxalate, and citrate), inflammation and uric acid metabolism. Safety endpoints include kidney function and tolerability. Adverse events were prospectively assessed at each study visit and safety call using prespecified clinical and laboratory criteria, with particular attention to ketoacidosis, genital or urogenital tract infection, and volume depletion or hypotension.

### Sample size

As no prior data were available to estimate the expected treatment effect of SGLT2 inhibition on divalent-ion handling in ADPKD, the sample size was determined pragmatically. Assuming a within-subject change of ∼10%–15%—corresponding to a moderate effect size (dz ≈ 0.5)—and 80% power for a two-period crossover design, we aimed to enroll 40 participants to allow for dropouts. Recruitment challenges across the 2 participating centers ultimately yielded 15 participants. Accordingly, the study was considered exploratory, and interpretation focused on estimation of effect sizes within 95% confidence intervals, safety and tolerability, rather than formal hypothesis testing.

### Statistical analysis

Analysis followed a per-protocol approach, appropriate for short mechanistic crossover trials and justified by the need to account for an allocation irregularity during enrollment. Continuous variables are presented as mean (standard deviation) or median (interquartile range), and categorical variables as counts and percentages.

Treatment effects were estimated using a generalized linear mixed-effects model (GLMM), with fixed effects for treatment and period and a random intercept for participant to account for within-subject correlation. Model estimates are reported as relative differences with 95% confidence intervals, and a two-sided α = 0.025. Statistical analyses were performed using R (version 4.5.0). Full model specifications and statistical analysis are described in the *Supplementary Material*.

## RESULTS

### Patient characteristics

Of 121 individuals screened (49 at KSGR and 72 at USZ), 16 were randomized and 15 completed both treatment periods (Fig. 1). The first participant was enrolled in October 2024, and the final visit occurred in July 2025. Baseline characteristics are provided in Table 1. The mean estimated glomerular filtration rate (eGFR) was 68 ml/min/1.73 m^2^, seven participants (47%) were on concomitant tolvaptan therapy, and none had diabetes mellitus. All completers were included in the per-protocol analysis.

**Figure 1:**
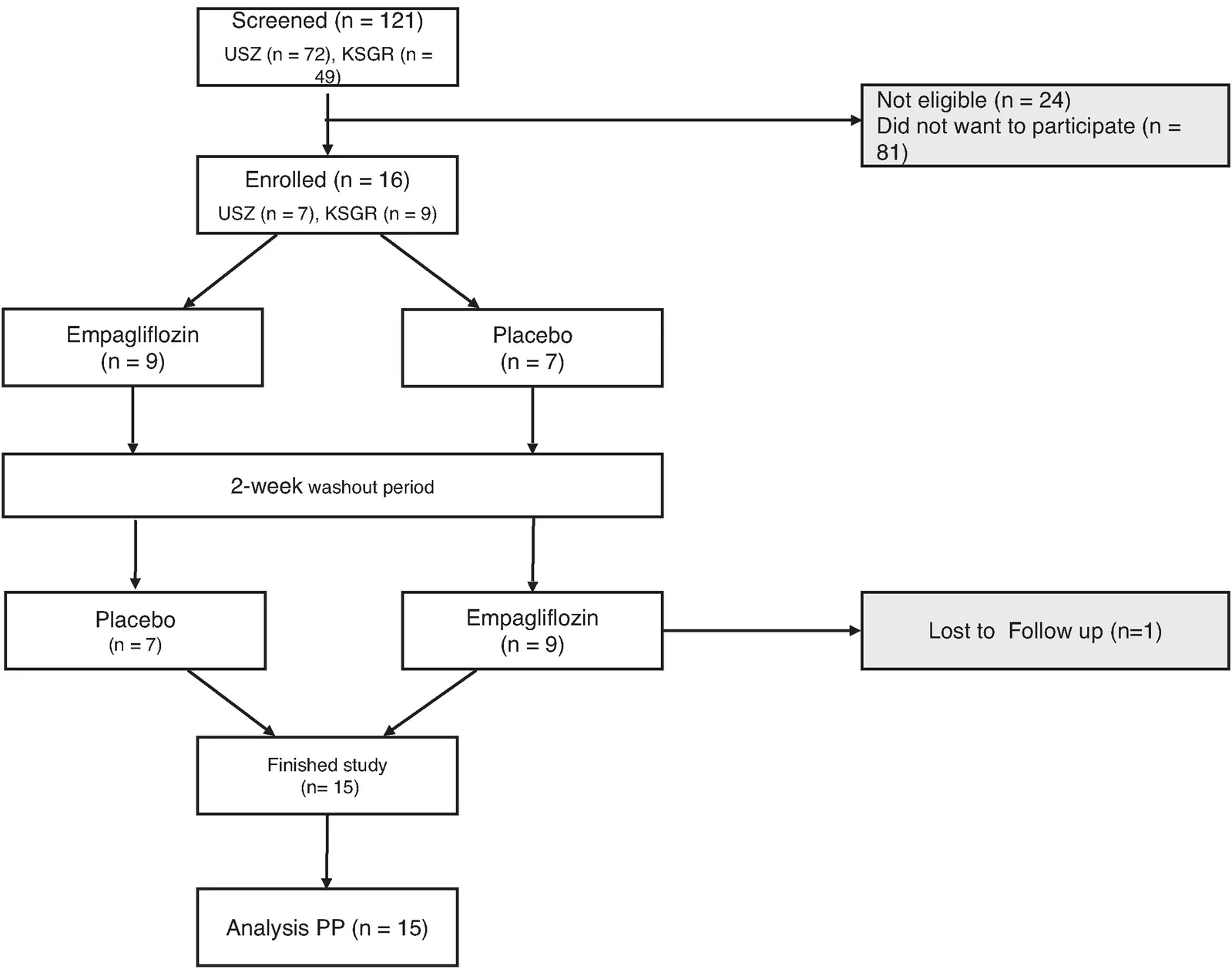
**Flow diagram of the SIDIA trial**.

**Table 1:** Baseline characteristics of participants.

Characteristics	Participants, *n* = 15
Age, years	43 [36, 52]
Sex, men (%)	8 (53)
Weight, kg	82 (21)
Height, cm	175 (9)
Body mass index, kg/m^2^	26.3 (7)
eGFR, ml/min/1.73 m^2^ body surface area	68 (20)
Mayo Imaging Classification (MIC)	
- 1A	3 (20)
- 1B	4 (26)
- 1C	4 (26)
- 1D	1 (7)
- 1E	3 (20)
Tolvaptan, *n* (%)	7 (47)
Hypertension	10 (67)
- Systolic blood pressure, mmHg	124 (10)
- Diastolic blood pressure, mmHg	78 (8)
Brain aneurysm, *n* (%)	3 (20)
Valvular disease, *n* (%)	0 (0)
Kidney stone, *n* (%)	1 (7)
Liver cysts, *n* (%)	10 (67)
Cyst complications, *n* (%)	2 (13)

Summary statistics of patient characteristics of study population. Categorical variables are shown as frequencies (%), continuous variables are described by their mean (standard deviation) or median [interquartile range]. Cyst complications are defined as hematuria and/or cyst infection. eGFR, estimated glomerular filtration rate.

### Primary outcomes

No substantial short-term differences in 24-h urinary or fractional excretion of phosphate, magnesium, or calcium were detected between empagliflozin and placebo (Fig. 2). Mean relative differences were small for phosphate (+10.8% [95% confidence interval (CI) −5.9 to +27.6]), calcium (−5.2% [95% CI −22.8 to +12.4]), and magnesium (+7.9% [95% CI -3.5 to +19.4]).

**Figure 2:**
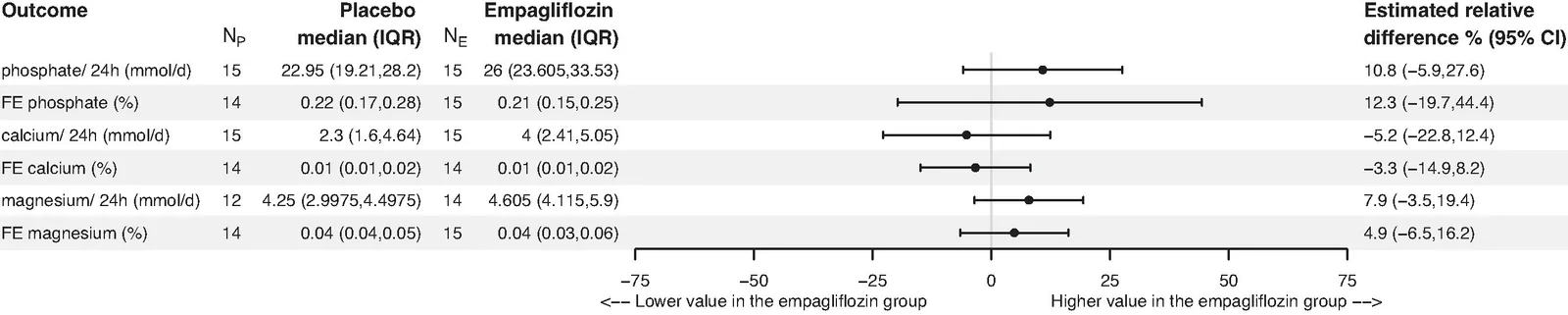
**Primary endpoint**. Forest plot of the effect of empagliflozin versus placebo on the primary endpoint. Medians of urinary excretions are shown, with first and third quartiles in parentheses. The dots in the forest plot represent the treatment effect on the respective urinary excretion as estimated relative difference (%), derived from a GLMM with a Gamma-distribution and a log link function with treatment and treatment period included as fixed effects and patient as a random intercept to account for the crossover design. Error bars represent associated 95% CIs. N_P_ Number of measurements assessed after Placebo treatment. N_E_ Number of measurements assessed after empagliflozin treatment. A two-sided significance level of 0.025 was used. Analyses are based on the full per-protocol cohort (n=15).

Unless otherwise specified, analyses were based on the full per-protocol cohort (*n* = 15). In sensitivity analyses restricted to participants who completed both treatment periods (complete crossovers; *n* = 11), estimates for the primary and secondary endpoints were consistent with the main analysis (Figs S4 and S5).

Carryover and sequence effects were formally assessed, with no evidence of either observed.

### Secondary and safety outcomes

Serum magnesium increased modestly during empagliflozin treatment compared with placebo (+5.7% [95% CI 3.5–8.0]), while serum uric acid decreased (-26% [95% CI -32.5 to -19.3]) and urinary citrate excretion rose (+78.6% [95% CI +52.7 to +104.4]) (Fig. 3). Urinary potassium excretion was also higher with empagliflozin (+17.4% [95% CI +1.8 to +33.0]), whereas other urinary electrolytes remained unchanged. Serum phosphate, calcium, and bone-mineral markers (PTH, FGF23, and 25-hydroxy-Vitamin D_3_) were unaffected. Serum creatinine increased slightly, with a corresponding transient decline in eGFR (-7.1% [-10.1 to -4.1]). Overall median urine volume was numerically higher with empagliflozin than with placebo (4.6 vs. 3.8 L). No changes were seen in inflammatory markers and liver function tests, and total urine volume also did not differ between the groups.

**Figure 3:**
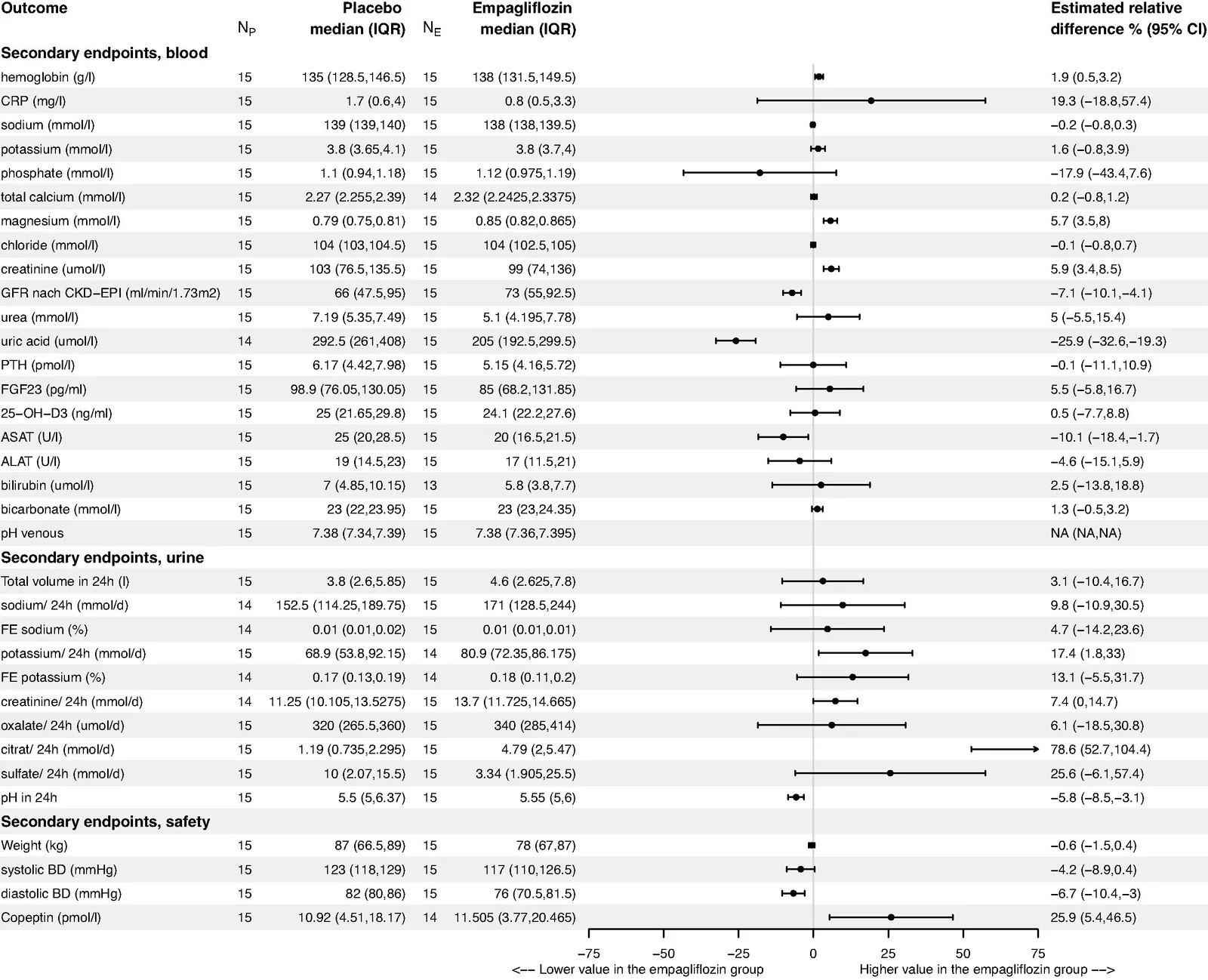
**Secondary and exploratory endpoints**. Forest plot of the effect of empagliflozin versus placebo on secondary endpoints. Medians are shown, with first and third quartiles in parentheses. The dots in the forest plot represent the treatment effect as estimated relative difference (%), derived from a GLMM with a Gamma-distribution and a log link function with treatment and treatment period included as fixed effects and patient as a random intercept to account for the crossover design. Error bars represent associated 95% CIs. N_P_ Number of measurements assessed after Placebo treatment. N_E_ Number of measurements assessed after empagliflozin treatment. Analyses are based on the full per-protocol cohort (n=15).

Empagliflozin was well tolerated, with a modest reduction in diastolic blood pressure while systolic blood pressure remained unchanged. Adverse events of particular interest—including genital infections, volume depletion, and ketoacidosis—were actively monitored; none occurred. Patient-reported side effects were generally mild and occurred with similar frequency under both treatments (Table S2).

Apparent differences between unadjusted means and GLMM estimates (for example, for eGFR) reflect model adjustment for period and within-participant correlation, providing a more accurate crossover-adjusted treatment effect.

### Subgroup analyses

In exploratory analyses stratified by concomitant tolvaptan therapy, empagliflozin had divergent effects on calcium handling. Among participants receiving tolvaptan, empagliflozin increased urinary calcium excretion (+22.6% [95% CI +11.1 to +34.2]), whereas in those not receiving tolvaptan, urinary calcium excretion decreased (−34.0% [95% CI −58.2 to −9.8]). Serum magnesium increased in both strata without evidence of interaction. Urinary phosphate and urinary magnesium excretion did not differ significantly between empagliflozin and placebo (Fig. 4).

**Figure 4:**
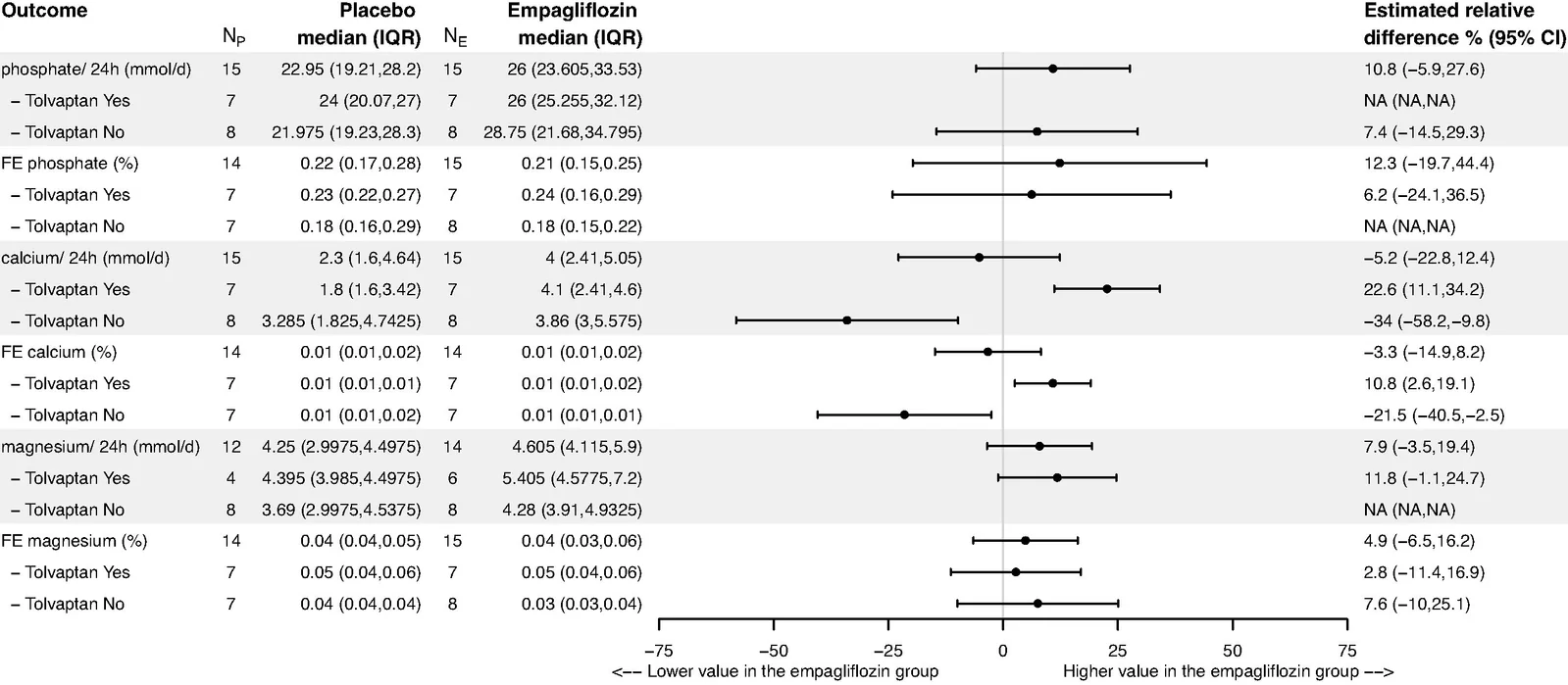
**Primary endpoint stratified by Tolvaptan**. Forest plot of the effect of empagliflozin versus placebo on the primary endpoint stratified by baseline tolvaptan use. Medians are shown, with first and third quartiles in parentheses. The dots in the forest plot represent the treatment effect as estimated relative difference (%), derived from a GLMM with a Gamma-distribution and a log link function with treatment and treatment period included as fixed effects and patient as a random intercept to account for the crossover design. Error bars represent associated 95% CIs. NP Number of measurements assessed after Placebo treatment. NE Number of measurements assessed after empagliflozin treatment. Analyses are based on the full per-protocol cohort (n=15).

For secondary endpoints, urinary potassium excretion was higher with empagliflozin in participants on tolvaptan (+22.8% [95% CI +1.3 to +44.2]), and urinary citrate excretion increased in both subgroups (+95.1% [95% CI +50.8 to +139.5]) (Fig. S2). No subgroup differences were observed for serum electrolytes, uric acid, or eGFR (Fig. S3). Median urine volume was similar with empagliflozin and placebo among participants not receiving tolvaptan (2.65 vs. 2.60 L) but showed a larger numerical increase among those receiving tolvaptan (8.6 vs. 6.0 L) (Fig. S2).

In a *post-hoc* analyses, copeptin concentrations were higher with empagliflozin than with placebo (+25.9% [95% CI +5.4 to +46.5]), with similar relative increases in participants receiving tolvaptan and those not on tolvaptan (Fig. 5).

**Figure 5:**
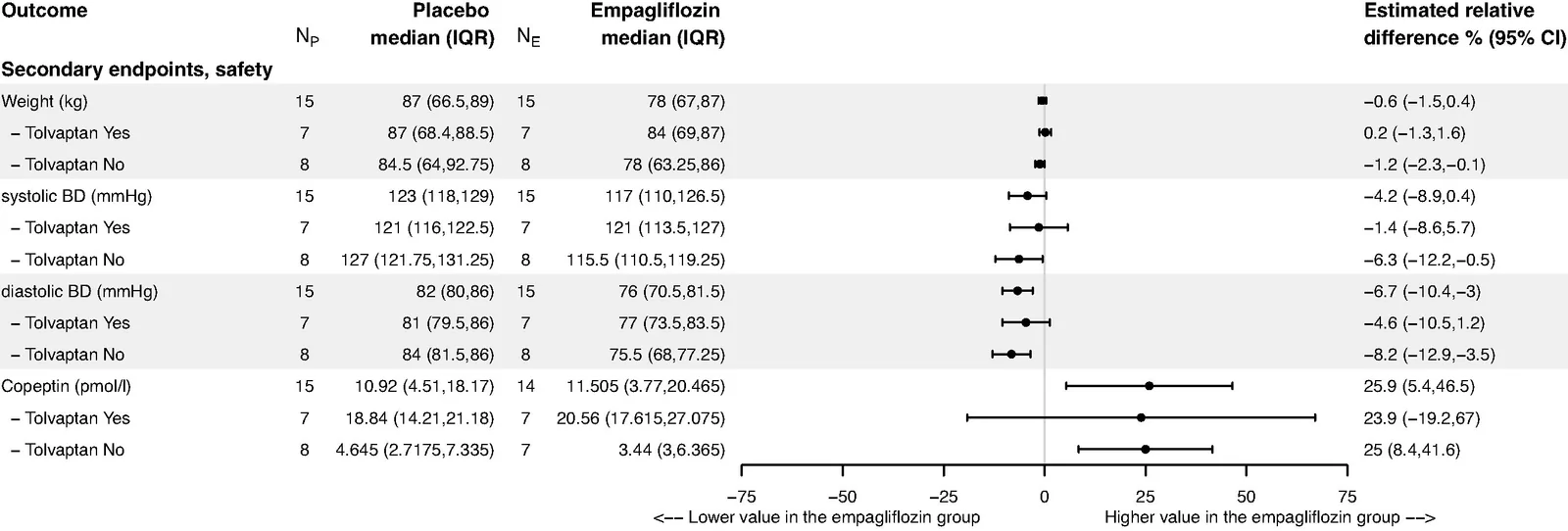
**Secondary and exploratory safety endpoints stratified by Tolvaptan**. Forest plot of the effect of empagliflozin versus placebo on secondary safety endpoints stratified by baseline tolvaptan use. Medians are shown, with first and third quartiles in parentheses. The dots in the forest plot represent the treatment effect as estimated relative difference (%), derived from a GLMM with a Gamma-distribution and a log link function with treatment and treatment period included as fixed effects and patient as a random intercept to account for the crossover design. Error bars represent associated 95% CIs. N_P_ Number of measurements assessed after Placebo treatment. N_E_ Number of measurements assessed after empagliflozin treatment. Analyses are based on the full per-protocol cohort (n=15).

## DISCUSSION

This randomized, double-blind, crossover trial provides the first controlled evaluation of SGLT2 inhibition in ADPKD, specifically focusing on divalent ion-handling and mineral metabolism. No substantial short-term differences in urinary phosphate, magnesium, or calcium excretion were detected between empagliflozin and placebo. Serum magnesium rose modestly, serum uric acid decreased, and urinary citrate excretion increased, whereas kidney function showed an expected transient decline in eGFR. No safety concerns or serious adverse events occurred, confirming the short-term metabolic stability, and good tolerability of empagliflozin in this population.

In line with prior SGLT2 studies in diabetes and CKD, only minor fluctuations in divalent-ion parameters were expected in this short mechanistic trial. The stability of phosphate, calcium, and magnesium excretion under empagliflozin contrasts with the modest changes reported in large diabetic and CKD cohorts, where SGLT2 inhibition transiently increases serum phosphate and magnesium through proximal NaPi-IIa/IIc transport and distal transient receptor potential melastatin 6 (TRPM6) activation [26, 27]. Such effects are typically detectable only in large populations and over longer exposures. In ADPKD, a disorder characterized by altered mineral metabolism, chronically elevated FGF23, and reduced Klotho expression may further blunt these tubular responses [24, 28]. The long-term impact of SGLT2 inhibitors on mineral bone disease (MBD) parameters remains uncertain; although a recent CKD study found lower risk of hyperphosphatemia and hypocalcemia and other favorable MBD profiles with SGLT2 inhibitor use [19, 29–31].

One speculative explanation is that tolvaptan, particularly when combined with empagliflozin, may influence calcium handling through complementary effects on tubular physiology. SGLT2 inhibition alters proximal sodium–glucose transport and downstream electrochemical gradients, which may secondarily affect calcium reabsorption, while tolvaptan-induced aquaresis increases tubular flow and reduces urine osmolarity, potentially modifying distal calcium handling. The interplay of these mechanisms may result in heterogeneous effects on urinary calcium excretion in ADPKD. However, these observations should be interpreted with caution, as this subgroup analysis is exploratory and the study was not powered for definitive conclusions.

Collectively, these short-term findings provide no evidence of a major disturbance in divalent-ion balance; however, smaller effects and longer-term changes cannot be excluded.

The rise in urinary citrate together with the reduction in serum uric acid represents the expected metabolic signature of SGLT2 inhibition, now confirmed in ADPKD. Hypocitraturia is common in this population and contributes to calcium-based nephrolithiasis, faster kidney growth, and is linked to disease progression [32–34]. In our study, hypocitraturia was common among participants (8/15 participants, 53%). As kidney function declines, proximal tubular citrate reabsorption increases as the kidney attempts to buffer the acid load, further lowering urinary citrate levels [32–34]. The substantial increase in citrate excretion observed here likely reflects reduced proximal-tubule reabsorption secondary to enhanced glucose and sodium delivery—an established effect of SGLT2 inhibition that may counteract this progressive hypocitraturia. SGLT2 inhibitors may therefore promote a less lithogenic urinary profile independent of urine pH, which remained unchanged in our study [35]. These findings align with findings from the SWEETSTONE trial, where empagliflozin increased urinary citrate and reduced biochemical stone-risk indices in non-diabetic kidney-stone formers [23].

Magnesium, a cofactor for adenosine triphosphate citrate synthase and an inhibitor of calcium crystallization, increased in the serum without a significant change in urinary excretion over the short study period. Prior work suggests that SGLT2 inhibitors can enhance distal magnesium reabsorption via epidermal growth factor-mediated TRPM6 activation in the distal convoluted tubule [36, 37]. The absence of a measurable urinary magnesium response in our study likely reflects the short 2-week treatment period, since the magnesium-raising effect of SGLT2 inhibitors typically develops gradually over several weeks and is most pronounced in individuals with baseline hypomagnesemia [37]. Higher magnesium concentrations are associated with greater urinary citrate excretion and reduced stone risk, suggesting a synergistic relationship that may become more pronounced with longer treatment [38]. Collectively, these findings suggest that SGLT2 inhibition may contribute to a less lithogenic profile in ADPKD; whether this translates into fewer stone events requires longer-term outcome studies. In addition, our findings are based on biochemical surrogates, as this trial did not assess clinical stone events or imaging endpoints given its short duration.

Beyond its effects on tubular transport and hemodynamics, SGLT2 inhibition triggers systemic metabolic adaptations that may be particularly relevant in ADPKD. By limiting proximal tubular glucose reabsorption, SGLT2 inhibitors reduce cellular glucose availability and shift energy metabolism toward fatty-acid oxidation and ketone utilization—pathways that oppose the glycolytic ‘Warburg-like’ phenotype described in cystic epithelia [39]. By enhancing metabolic efficiency, inducing mild ketosis, and modulating key signaling pathways such as activated protein kinase (AMPK) and mammalian target of rapamycin (mTOR), SGLT2 inhibitors may help reduce cellular stress and support kidney protection [40, 41]. In a *post-hoc* analysis of a large ADPKD cohort, greater visceral adiposity was associated with faster kidney growth and disease progression, underscoring the link between metabolic status and cyst expansion [42]. Consistent with this concept, the RESET-PKD pilot trial demonstrated that short-term ketogenic interventions successfully induced ketogenesis and reduced total liver volume, highlighting the potential value of metabolic reprogramming [43]. These external observations provide a biological rational for studying metabolic interventions in ADPKD. However, SIDIA primarily provides short-term data on metabolic and mineral handling and did not assess direct effects on cyst growth or kidney or liver volumes. Whether SGLT2 inhibition modifies these structural outcomes requires dedicated longer-term trials with imaging endpoints.

No major short-term safety signals or serious adverse events were observed in this small mechanistic trial, although the exploratory increase in copeptin warrants further evaluation. Kidney function showed a modest decline in eGFR, consistent with the well-described hemodynamic dip observed with SGLT2 inhibition, which is generally considered benign and may even predict long-term renal benefit through restoration of tubuloglomerular feedback [44]. No clinically relevant changes occurred in blood pressure or hydration status, supporting the short-term safety of SGLT2 inhibitors in ADPKD. Recent data from the first completed open-label, randomized trial in ADPKD similarly demonstrated that dapagliflozin was safe and well-tolerated over 6 months and did not accelerate eGFR decline or kidney-volume growth, reinforcing the favorable short-term safety profile of SGLT2 inhibition in this population [40].

Nevertheless, two key caveats merit consideration. First, the higher urine volume appeared to be driven by participants receiving concomitant tolvaptan, whereas urine volume remained essentially unchanged among those not receiving tolvaptan. Although based on small exploratory subgroups, this finding suggests that empagliflozin-induced osmotic diuresis may augment tolvaptan-induced aquaresis. Second, the exploratory *post-hoc* finding of higher copeptin raises a potential concern, but warrants confirmation. Vasopressin signaling promotes cyst growth in ADPKD and may counteract the potential benefits of SGLT2 inhibition [45]. Similar increases in copeptin following SGLT2 inhibitor initiation have been reported in patients with type 2 diabetes and heart failure and may reflect an adaptive vasopressin-mediated response to glucosuria-induced osmotic diuresis [45, 46]. Whether this adaptive response persists with longer exposure or in combination with V_2_-receptor antagonism remains uncertain [47]. Future trials should therefore include systematic longitudinal monitoring of copeptin.

Several larger randomized controlled trials (NCT05510115, NCT06391450) are currently underway to assess the long-term efficacy and safety of SGLT2 inhibitors in ADPKD. The present findings reinforce feasibility and short-term safety while identifying mechanistic questions for future studies.

Limitations include the small sample size and short treatment duration. Recruitment challenges led to early termination after 15 participants completed the study, below the planned 36 completers, limiting statistical power and precision. Accordingly, the analyses were exploratory, and smaller treatment effects cannot be excluded. Nevertheless, the randomized crossover design, standardized sampling protocol, and consistent results across sensitivity analyses strengthen internal validity.

In summary, no substantial short-term differences in divalent-ion handling were detected with empagliflozin in ADPKD, while improving urinary citrate and uric acid profiles. These data support further evaluation of SGLT2 inhibitors as adjunctive therapy in ADPKD and provide a physiological framework for interpreting outcomes of ongoing long-term trials.

## Supplementary Material

sfag349_Supplemental_File

## Data Availability

The data underlying this article are available in the article and in its online supplementary material.
