# Supplementary material for: Short-term effects of an SGLT2 inhibitor on divalent ions in autosomal dominant polycystic kidney disease: a randomized crossover trial
Source: Clin Kidney J. 2026 Oct 2;19(10):sfag349. doi: 10.1093/ckj/sfag349 (PMC13696889; doi:10.1093/ckj/sfag349)
Supplement: Supplementary file 1 [file sfag349_supplemental_file.docx]

**SUPPLEMENTARY**

**SUPPLEMENTARY METHODS**

**Study Design and Oversight**

The SIDIA trial (Short-term effects of an SGLT2 inhibitor on divalent ions in autosomal dominant polycystic kidney disease) was an investigator-initiated, randomized, double-blind, placebo-controlled, two-period crossover mechanistic study conducted at two Swiss academic centers: the KSGR and USZ. Each treatment period lasted two weeks and was separated by a two-week washout.

The trial was designed and sponsored by the investigator team at KSGR (Sponsor–Investigator: Prof. Dr. Thomas Fehr) in collaboration with USZ. Dr. Patrick Hofmann and dipl. med. Lorena Roth served as co-investigators and study coordinators. The clinical protocol (Version 2.0, 23 May 2024) governed all study activities.

*Ethical and regulatory approval*

The study was authorized by Swissmedic (Authorization No. 701827; approval 10 June 2024) and approved by the Cantonal Ethics Committee Zurich (BASEC ID 2024-00070). It was categorized as a risk category B clinical trial under the Swiss Human Research Act (HFG) and the Ordinance on Clinical Trials (ClinO). The trial was prospectively registered in the Swiss National Clinical Trials Portal (SNCTP000005836) and on ClinicalTrials.gov (NCT06435858) before participant enrolment.

All procedures complied with the **Declaration of Helsinki (2013), ICH-GCP E6(R2),** and applicable Swiss laws. Written informed consent was obtained from all participants before any study-related procedure. Study conduct and documentation were monitored by the Clinical Trial Unit KSGR according to a predefined monitoring plan. Because of the short duration, low risk, and use of an approved drug at standard dose, a data-safety monitoring board was not required.

**Participants**

Adults aged 18 to 75 years with a diagnosis of autosomal dominant polycystic kidney disease (ADPKD) established by internationally accepted imaging criteria (KDIGO 2015) were eligible for enrollment. Participants were recruited consecutively from nephrology outpatient clinics at KSGR and USZ.

Inclusion criteria were:

- Patients 18-75 years old with ADPKD, defined according to international diagnostic and classification criteria^1^, treated at KSGR and USZ independent of baseline treatment with the vasopressin receptor antagonist Tolvaptan
- Informed consent as documented by signature

Exclusion criteria were:

- renal replacement therapy or kidney allograft recipient
- chronic kidney disease CKD KDIGO Stage G4 (eGFR < 30ml/min/1.73m^2^)
- patients < 18 years of age
- Diabetes mellitus type 1
- recurrent urinary tract infections (UTI) defined as ≥3 infections requiring antibiotic treatment or ≥1 requiring hospitalization/year.
- Patients with uncontrolled hypertension (defined as ambulatory systolic BP > 180 fmmHg), liver cirrhosis (Child Pugh B&C)
- Patients not able or not willing to stop the following medications during the study period of participation in the trial:
  - Thiazide diuretics
  - Carbonic anhydrase inhibitors
  - Sodium bicarbonate
  - 1, 25 (OH) vitamin D (calcitriol)
  - Bisphosphonate, denosumab, teriparatide
- Pregnant or lactating women
- Known allergy to study drug

Eligible individuals received oral and written study information and had at least 24 hours to decide about participation. The informed-consent form was signed by the participant and an investigator before any protocol-specific procedure. No vulnerable populations were included, and no financial incentives were offered.

**Randomization, Masking, and Interventions**

*Randomization*

Participants were randomized in a 1:1 ratio to one of two treatment sequences (empagliflozin followed by placebo, or placebo followed by empagliflozin) using a computer-generated randomization list ([www.randomizer.at](http://www.randomizer.at)).

The trial followed a double-blind design with patients and treating physicians blinded to treatment allocation and laboratory data, whereas laboratory team and biostatistician were unblinded. Full double-blinding was not pursued because the presence of glucosuria allows reliable identification of SGLT2 inhibition during laboratory analysis, and masking this effect would have required disproportionate procedural complexity for a mechanistic study.

Each treatment period lasted two weeks, separated by a two-week washout. Participants took one capsule daily in the morning, independent of food.

*Intervention/investigational product*

The investigational product was empagliflozin (Jardiance® 10 mg, Boehringer Ingelheim), a beige, round tablet containing empagliflozin and lactose monohydrate. To ensure visual blinding, each tablet was enclosed in an opaque hard gelatin capsule. The control product consisted of a matching hard gelatin capsule containing a “P-Tabletten weiss 8 mm Lichtenstein®” tablet (lactose monohydrate, cellulose powder, magnesium stearate) provided by the Hospital Pharmacy of the University Hospital Basel. This approach produced identical appearance and handling between active and placebo capsules.

Both empagliflozin and placebo capsules were packaged in glass containers (14 capsules per container), labelled, and blinded by the hospital pharmacy of the University Hospital Basel. Study medication was stored in a locked facility with restricted investigator access at room temperature (15–25°C).

Adherence was assessed by capsule count and participant self-report at each visit. Participants were instructed to maintain adequate hydration and to temporarily discontinue study medication in case of acute illness, vomiting, diarrhea, or dehydration (“sick-day rule”) and immediately contact their corresponding study site.

Due to dispensing logistics, four participants received the same treatment in both periods. These participants were included in the per-protocol analysis, and sensitivity analyses excluding them yielded similar results.

**Study Procedures**

*Clinical assessments*

Each participant completed a seven-week study sequence consisting of three in-person visits (baseline, end of period 1, end of period 2) and two interim safety phone calls. The overall design included two two-week treatment periods separated by a two-week washout.

At each in-person visit, a standardized exam including assessment of vitals was performed, medication was reviewed, and adverse-event information was collected. Female participants of child-bearing potential underwent urine pregnancy testing before inclusion in the study.

At each visit and safety call, participants completed a brief questionnaire on tolerability, thirst, dizziness, urinary frequency, and fluid intake. Investigators documented predefined adverse events of special interest (volume depletion, signs or symptoms of urinary tract infection, hepatic enzyme elevation, and hypotension) in the case-report form. All events were reviewed by the principal investigator for seriousness and relatedness. *Supplementary Figure S1*

*Sample collection*

Participants performed a 24-hour urine collection before each visit. Completeness was verified by urinary creatinine and urine volume. Venous blood samples were obtained after completion of the urine collection. All handling followed standardized operating procedures approved by the study sponsor and consistent with Good Clinical Practice (GCP).

*Analytes*

Serum analyses included electrolytes (Na⁺, K⁺, Cl⁻, Ca²⁺, Mg²⁺, PO₄³⁻), creatinine, urea, uric acid, PTH, FGF23, 25-hydroxy-vitamin D₃, liver enzymes (AST, ALT, bilirubin), venous blood gas (vBGA), C-reactive protein and copeptin.

Urine analyses comprised volume, osmolarity, pH, creatinine, calcium, phosphate, magnesium, sodium, potassium, citrate, sulfate, oxalate, and ketones.

**Laboratory Analysis**

All laboratory analyses were performed in accredited clinical laboratories at KSGR and USZ according to institutional standard operating procedures using standard laboratory methods in line with recent recommendations.^50^ Urine collections were performed under paraffin oil with thymol as an additive. Urine pH was measured by an electrode pH meter calibrated daily. Fractional excretions were calculated as:

$$FE_{x}=\frac{U_{x}\times S_{creat}}{S_{x}\times U_{creat}}\times100\%$$

where x represents, phosphate, magnesium, or calcium. Estimated glomerular filtration rate (eGFR) was derived from serum creatinine using the CKD-EPI 2021 equation.

All analyses were performed according to GCP and the approved study protocol (Version 2.0, May 2024). Laboratory staff and biostatistician were not blinded to treatment allocation.

**Outcomes**

The primary outcome was the effect of empagliflozin compared with placebo on renal tubular handling of divalent ions—phosphate, magnesium, and calcium—assessed by 24-hour urinary excretion and corresponding fractional excretion values.

Secondary and exploratory outcomes included tubular handling of other electrolytes (sodium, potassium, and chloride), and markers of mineral and bone metabolism (PTH, FGF23, and 25-hydroxy-vitamin D). Additional exploratory outcomes comprised urinary parameters (volume, pH, oxalate, and citrate), markers of inflammation, and indices of uric acid metabolism.

Safety endpoints included kidney function (serum creatinine, eGFR) and tolerability, assessed through systematic recording of adverse events and patient-reported symptoms at each study visit and safety call.

**Statistical Analysis Plan**

*Sample size rationale*

No prior data existed on SGLT2 inhibitor effects on divalent-ion handling in ADPKD. The assumed within-subject change of 10-15% was extrapolated from studies in patients with diabetes treated with SGLT2 inhibitors.^51^  Based on this expected effect and an estimated standard deviation of 20%, a two-period crossover design with 80% power and α = 0.025 yielded a target of 40 participants (36 completers). Recruitment feasibility limited enrolment to 15 completers; results are therefore interpreted as exploratory, focusing on effect-size estimation with 95% confidence intervals.

*Statistical Analysis*

Analyses followed a per-protocol approach because the objective of this short mechanistic trial was to estimate the physiological effects of treatment received among participants who completed both study periods. All participants who completed both study periods were included, including the four who, due to site-level dispensing irregularities, received the same treatment in both periods (two placebo-placebo, two empagliflozin-empagliflozin). Their data were retained to preserve sample size, and sensitivity analyses excluding these participants yielded consistent results, compare figures S4 and S5 against figure 2 and figure 3.

Continuous variables were expressed as mean ± SD or median (IQR) and categorical variables as counts (%). Generalized linear mixed-effects models (GLMMs) were used for continuous outcomes, with fixed effects for treatment and period (1 = treatment first, 0 = both periods same, -1 = placebo first) and a random intercept for participants to account for within-subject correlation. Because GLMMs can accommodate unbalanced data and missing crossover sequences, measurements from all participants-including those without treatment alternation-could validly contribute to estimation of treatment effects through the within subject information. Model diagnostics included inspection of residual distributions, models that failed to converge flagged and reported as NA in the figures, and robustness was assessed through sensitivity analyses restricted to complete crossovers. Model results are reported as relative difference with 95% confidence intervals and a two-sided alpha = 0.025. All analyses were performed in R version 4.5.0. Anonymized data, diagnostic plots, residual distributions, and analysis code are available on request.

**Safety Monitoring**

Adverse events were collected systematically at each visit and via safety calls. Events of special interest included:

- euglycemic ketoacidosis (pH < 7.25, HCO₃⁻ < 14 mmol/L),
- hepatic injury (ALT > 3× baseline),
- symptomatic urinary tract infection,
- dehydration or hypotension (SBP < 100 mmHg), and
- polycythemia (hematocrit > 49% men / 48% women).

Serious AEs (SAEs) were to be reported within 24 hours to the Sponsor-Investigator and to Swissmedic via BASEC if applicable. No SAEs or SUSARs occurred during the trial.

**Quality Assurance and Data Handling**

All source data were entered into a pseudonymized electronic case-report form (Excel-based, password-protected, stored on secure KSGR server). Only authorized investigators had access. Regular backups and version control were implemented.

Study documents will be archived for ≥ 10 years after study completion in accordance with Swiss law. Monitoring was conducted by the CTU KSGR under GCP standards. Data inspection and audit rights were granted to Swissmedic and the Ethics Committee.

**Insurance and Funding**

Study insurance was provided by Zurich Insurance AG (contract nr. 16.420.693) in compliance with Swissmedic and local ethics committee. The trial was funded by the Vontobel Foundation (Zurich), Boehringer Ingelheim and AstraZeneca. The sponsor-investigator retained full independence over design, data analysis, and publication. No involvement from industry occurred in data handling or manuscript preparation.

**Data Availability**

Anonymized data and the statistical analysis code (R-script) are available from the corresponding author upon request and in accordance with institutional data-sharing policies.

**CONSORT Compliance**

This study was conducted and reported in accordance with the new CONSORT 2025 statement for randomized crossover trials. ^25^

A detailed participant flow diagram is provided in *Figure 1.*

**SUPPLEMENTARY TABLES & FIGURES**

**SUPPLEMENTARY FIGURE LEGENDS**

**Figure S1. Study Crossover Flow Diagram**

**Figure S2. Secondary and exploratory urinary endpoints stratified by tolvaptan**

Forest plot of the effect of empagliflozin versus placebo on secondary urinary endpoints stratified by baseline tolvaptan use. Medians are shown, with first and third quartiles in parentheses. The dots in the forest plot represent the treatment effect as estimated relative difference (%), derived from a GLMM with a Gamma-distribution and a log link function with treatment and treatment period included as fixed effects and patient as a random intercept to account for the crossover design. Error bars represent associated 95% CIs. N_P_ Number of measurements assessed after Placebo treatment. N_E_ Number of measurements assessed after empagliflozin treatment. Analyses are based on the full per-protocol cohort (n=15).

**Figure S3. Secondary and exploratory serum endpoints stratified by tolvaptan**

Forest plot of the effect of empagliflozin versus placebo on secondary serum endpoints stratified by baseline tolvaptan use. Medians are shown, with first and third quartiles in parentheses. The dots in the forest plot represent the treatment effect as estimated relative difference (%), derived from a GLMM with a Gamma-distribution and a log link function with treatment and treatment period included as fixed effects and patient as a random intercept to account for the crossover design. Error bars represent associated 95% CIs. N_P_ Number of measurements assessed after Placebo treatment. N_E_ Number of measurements assessed after empagliflozin treatment. Analyses are based on the full per-protocol cohort (n=15).

**Figure S4. Sensitivity analysis of the primary endpoint restricted to participants with complete crossover exposure**
Forest plot of the effect of empagliflozin versus placebo on primary endpoints, restricted to participants with complete crossover exposure. Treatment effects are shown as estimated relative differences (%) with 95% confidence intervals derived from GLMMs with treatment and period as fixed effects and participant as a random intercept to account for the crossover design. Error bars represent associated 95% CIs. N_P_ Number of measurements assessed after Placebo treatment. N_E_ Number of measurements assessed after empagliflozin treatment. Analyses are restricted to participants who received both treatments (complete crossovers; n=11).

**Figure S5. Sensitivity analysis of secondary endpoints restricted to participants with complete crossover exposure**
Forest plot of the effect of empagliflozin versus placebo on secondary endpoints, restricted to participants with complete crossover exposure. Treatment effects are shown as estimated relative differences (%) with 95% confidence intervals derived from GLMMs with treatment and period as fixed effects and participant as a random intercept to account for the crossover design. Error bars represent associated 95% CIs. N_P_ Number of measurements assessed after Placebo treatment. N_E_ Number of measurements assessed after empagliflozin treatment. Analyses are restricted to participants who received both treatments (complete crossovers; n=11).

**SUPPLEMENTARY TABLES**

**Supplementary Table S1. Baseline Medications Prior to Study Initiation**

| **Medication Class** | **Overall Cohort (n=15)** |
| --- | --- |
| RAAS inhibitor | 13 (87) |
| Tolvaptan* | 7 (47) |
| Calcium antagonist | 7 (47) |
| Statin | 4 (27) |
| Thiazides† | 2 (13) |
| Uricosurics | 2 (13) |
| SNRI/SSRI | 2 (13) |
| Cholecalciferol (vitamin D3) | 1 (7) |
| Mineralocorticoid receptor antagonist | 1 (7) |
| Beta blocker | 1 (7) |
| Metformin | 1 (7) |
| Octreotide | 1 (7) |

Values are presented as n (%). Participants could receive more than one medication.

*All seven patients were treated with 120mg daily.

†Thiazides were discontinued two weeks before study initiation according to study exclusion criteria.

**Supplementary Table S2. Adverse events during the study period**

| **Adverse Event (AE)** | **Active Treatment (n=15)** | **Control (n=15)** |
| --- | --- | --- |
| Any AE | 7 (47) | 7 (47) |
| Serious AE | 0 | 0 |
| Discontinuation due to AE | 0 | 0 |
| Exanthema | 1 (7) | 1 (7) |
| Diarrhoea | 2 (13) | 1 (7) |
| Nausea | 2 (13) | 1 (7) |
| Fever | 2 (13) | 2 (13) |
| Dizziness | 2 (13) | 1 (7) |
| Thirst | 0 | 1 (7) |

Values represent the number (percent) of participants experiencing the event. A participant may experience more than one event.
